# Persuasive differences between human and virtual influencers in health supplement advertising: evidence from eye-tracking

**DOI:** 10.3389/fpsyg.2025.1692737

**Published:** 2026-01-06

**Authors:** Mengqi Du, Kyung Han You (Ryu)

**Affiliations:** Department of Media and Communication Studies, Jeonbuk National University, Jeonju, Republic of Korea

**Keywords:** advertising effectiveness, virtual influencers, authenticity, human-likeness, processing fluency, health supplement products, eye-tracking

## Abstract

**Introduction:**

Attention is scarce in fast-paced, visually saturated feeds—especially for trust-sensitive categories such as health supplements, where credibility relies on source cues. Eye-tracking shows how viewers engage with advertising elements, yet authenticity and perceived human-likeness vary between human and virtual endorsers. We posit that source type moderates whether attention translates into evaluation. To examine these effects, we investigate whether source type influences effectiveness, whether attention drives outcomes, and whether these relationships depend on source type.

**Methods:**

In a laboratory experiment with an East Asian university student sample (*N* = 120), an Instagram-style vitamin C advertisement was displayed using a 2 × 2 design (human vs. virtual influencer; gender). Gaze was tracked with Tobii Pro Nano for three areas: the endorser’s face, the product, and the text. After viewing, participants rated their attitude toward the advertisement and purchase intention. Hierarchical regressions with interactions were used to test main and moderating effects.

**Results:**

There are persuasive differences between human and virtual influencers. Virtual influencers tended to attract more visual attention, but this did not consistently correspond to more favorable outcomes. By contrast, human influencers were generally associated with more positive advertising attitudes and stronger purchase intentions. Attention to text and face appeared to be predictors of advertising effectiveness. Importantly, source type appeared to moderate the link between attention and outcomes: for human endorsers, greater face attention was associated with more favorable evaluations, whereas for virtual endorsers, similar levels of attention were not accompanied by comparable gains.

**Discussion:**

The findings shift focus from “who performs better” toward understanding “when and why” attention persuades. Attention influences persuasion through a processing fluency-based mechanism, operating via the ease with which the source is perceived as human—both visually and mentally. Eye-tracking complements self-reports by revealing processing before conscious awareness. The patterns yield preliminary hypotheses that human endorsers may better support credibility-intensive claims and suggest a testable framework for how attention could translate into persuasion, offering a starting point for future targeted evaluations.

## 1 Introduction

The rise of digital production technologies has fueled new forms of brand collaboration with influencers, including the growing phenomenon of virtual influencers. Virtual influencers are computer-generated endorsers—CGI/AI-based personas managed by brands or creators—whose appearance, speech, and narratives are fully programmable (Kim and Wang, 2023; Zeng et al., 2024; Audrezet et al., 2025). They enable always-on availability and fine-grained, centralized control over identity and messaging; their scalability and perceived brand safety allow rapid creative iteration, supporting data-driven campaign optimization at lower operational risk and cost (Conti et al., 2022; Gerlich, 2023; Gulan et al., 2025).

Building on these capabilities, collaborations between virtual influencers and brands increasingly extend beyond fashion and entertainment into the health domain (Brachtendorf, 2022; Chung et al., 2024; Ju et al., 2024). For example, Rozy—a virtual influencer created by the South Korean startup Sidus Studio X—has drawn attention by promoting health supplements. These practices heighten consumer concerns about the credibility of virtual influencers, as not all endorsers possess the necessary expertise to communicate accurate health information (Díaz-Martín et al., 2020). Because virtual influencers lack real-world health experience and verifiable embodiment, they widen the perceived authenticity gap and may intensify consumer uncertainty (Franke et al., 2023; Lou et al., 2023; Belanche et al., 2024). To better understand why such credibility gaps emerge, it is necessary to consider how people psychologically relate to nonhuman agents in mediated environments.

Recent insights from Human–Computer Interaction (HCI) research provide a useful lens for these concerns. Studies have shown that people interact with computer-generated agents in systematically different ways than they do with human communicators, which shapes perceptions of trust (Sundar and Kim, 2019; Hong et al., 2020). Virtual influencers are often perceived as less authentic (Lou et al., 2023; Liu and Lee, 2024) and less transparent (Kim and Wang, 2023; González-Díaz et al., 2024; Khalfallah and Keller, 2025). They are also viewed as having lower visual and mental human-likeness (Mo and Zhou, 2024; Stein et al., 2024; Liu et al., 2025; Olbermann and Nagl, 2025), which can evoke “uncanny” reactions and increase skepticism (Lou et al., 2023; Gutuleac et al., 2024). Nonetheless, some studies suggest that virtual influencers perform as well as, or even better than, human influencers in certain contexts (Belanche et al., 2024; Mo and Zhou, 2024). Overall, the evidence remains mixed, and it is still uncertain whether these patterns extend to the promotion of health supplements. Because health supplements are credence goods, whose efficacy is often difficult to verify, consumers tend to rely on trusted and expert sources to reduce uncertainty (Andersen, 1994; Nagler et al., 2011). This raises the boundary-condition question of whether virtual influencers can credibly and persuasively endorse such products.

Methodologically, most studies rely on self-reported measures, which provide limited insight into the cognitive dynamics of how audiences process visually rich social media advertising (Byun and Ahn, 2023; Franke et al., 2023; Belanche et al., 2024; Dondapati and Dehury, 2024; Zhou et al., 2024). Eye-tracking provides an objective way to measure visual attention, a key factor shaping brand awareness, attitudes, and purchase intentions (Kumar and Gupta, 2016; Febriyantoro, 2020). By recording gaze patterns, it identifies elements that attract attention (Chen et al., 2022; Jánská et al., 2024). Comparing gaze responses to human versus virtual influencers can reveal implicit preferences and motivational mechanisms beyond self-report data (Białowąs and Szyszka, 2019; Pozharliev et al., 2022).

To our knowledge, no study has directly compared human and virtual influencers in promoting health supplements through eye-tracking. This exploratory experiment therefore examines how influencer type and visual attention jointly shape the effectiveness of health supplement advertising, and whether attention to virtual influencers predicts outcomes in a manner comparable to human influencers. The study’s main contributions are: (1) clarifying how attention drives persuasion across different types of influencers; (2) enhancing measurement accuracy through objective attention metrics; and (3) offering tentative, evidence-informed insights for endorser selection, serving as a starting point for future empirical validation and normative discussion.

## 2 Literature review

### 2.1 Source credibility and perceived authenticity in health endorsement

In health-related endorsements, source credibility and perceived authenticity are distinct yet interrelated constructs that jointly influence consumer responses. Source credibility refers to the audience’s evaluation of an endorser’s expertise, trustworthiness, and attractiveness (Ohanian, 1990). In contrast, perceived authenticity concerns the endorser’s genuineness, transparency, and perceived personal experience (Lee and Eastin, 2021). Authenticity has been recognized as a critical determinant of consumer trust and persuasive effectiveness (Audrezet et al., 2020; Lee and Eastin, 2021; Hasan et al., 2024). In this study, authenticity is conceptualized as a precursor to credibility, as endorsers perceived as more authentic are likely to be viewed as more credible, thereby enhancing persuasive impact (Han and Balabanis, 2024).

Health supplement marketing faces a unique challenge because such products are credence goods, whose efficacy and safety are difficult to verify even after consumption, which makes consumers rely more on trustworthy diagnostic cues (Darby and Karni, 1973; Andersen, 1994). In high uncertainty contexts, authenticity becomes a key factor in reducing uncertainty and supporting credible judgments (Lee, 2020; Park et al., 2022). However, not all authenticity cues provide equal evidentiary value. According to warranting theory, information carries greater warranting value when it is less subject to manipulation by the person it describes (Walther and Parks, 2002; DeAndrea, 2014). Thus, cues linked to an endorser’s verifiable offline reality are perceived as more reliable than those from computer-generated agents. Building on this logic, authenticity cues grounded in real-life experiences are more likely to foster trust and perceived credibility. Audiences therefore tend to trust human influencers who demonstrate genuine engagement in health practices and share credible, personal testimonials about product effectiveness and safety (Pilgrim and Bohnet-Joschko, 2019; Dondapati and Dehury, 2024).

Human influencers can provide high-warrant evidence through self-disclosure, coherent health narratives, and visible lifestyle choices (Pilgrim and Bohnet-Joschko, 2019; Christiansen et al., 2025; Ye and Li, 2025). Such embodied authenticity cues reduce ambiguity and enhance perceived diagnosticity, leading to more favorable evaluations of both the advertisement and the endorsed brand, and ultimately to stronger purchase intentions and behaviors (Agnihotri et al., 2023; Liu and Zheng, 2024; Hasan et al., 2024).

By contrast, virtual influencers often face a perceived authenticity deficit, especially in risk-sensitive health contexts (Khalfallah and Keller, 2025; Belanche et al., 2024). Lacking real-life experiences and verifiable health narratives, virtual influencers may struggle to establish authenticity and build parasocial relationships, which weakens their perceived credibility and persuasive impact (Lou et al., 2023; Franke et al., 2023). Although their novelty and controllability may be advantageous in utilitarian product categories, these traits often backfire in sensitive or high-risk contexts, such as health-related communication (Belanche et al., 2024). Moreover, when audiences cannot easily recognize the influencer as artificial, issues of transparency and misidentification may arise, increasing skepticism toward both the influencer and the message (Kim and Wang, 2023). Taken together, these insights suggest a persuasion advantage for human over virtual endorsers in health-related contexts. Accordingly, we hypothesize:

*H1a:* Human influencers elicit more favorable attitudes toward health supplement advertisements than virtual influencers.

*H1b:* Human influencers generate higher purchase intentions for health supplements than virtual influencers.

### 2.2 Visual attention and influencer advertising: mechanisms of persuasive impact

Visual attention plays a central role in consumer engagement with advertisements, linking exposure to persuasive outcomes (Pieters and Wedel, 2004). It provides an immediate and measurable indicator of interest and cognitive involvement (Pieters and Wedel, 2004; Kumar and Gupta, 2016), thereby clarifying how advertising shapes consumer responses. Eye-tracking, an unobtrusive method for capturing gaze behavior, reveals how attention is distributed across advertisement elements and connects these patterns to persuasive outcomes in realistic viewing contexts (Rayner, 1998; Wedel and Pieters, 2008; Holmqvist et al., 2011; Duchowski, 2017). Thus, eye-tracking is an valuable tool for examining how visual attention during advertising exposure shapes later evaluations.

Key advertisement elements produce distinct effects on gaze allocation, shaped by both bottom-up stimulus features and top-down goals or expectations (Yarbus, 1967; Henderson, 2003; Wedel and Pieters, 2008; Borji and Itti, 2013). According to biased competition theory, visual information competes for limited processing capacity, and both task goals and stimulus characteristics guide which elements receive deeper processing (Desimone and Duncan, 1995). Based on this framework, we track eye movements toward three common elements in influencer advertising—product, brand, and endorser—to isolate each element’s contribution to persuasion.

Endorsers’ faces are privileged social cues that capture attention quickly and enhance perceived credibility. They help direct focus toward message-relevant content, improving memory and overall advertising evaluation (Haxby et al., 2000; Cerf et al., 2009; Sajjacholapunt and Ball, 2014; Adil et al., 2018; Guido et al., 2018). Longer fixations on endorsers are associated with more favorable brand attitudes and stronger purchase intentions across both print and audiovisual contexts (Adil et al., 2018; Zhang and Yuan, 2018).

Product visuals anchor the advertisement by drawing attention to concrete features and aligning evaluation processes with advertising goals. Increased viewing time on key product areas enhances product evaluations and purchase intentions (Pieters and Warlop, 1999; Wedel and Pieters, 2008; Chandon et al., 2009; Atalay et al., 2012; van Loon et al., 2022). In both traditional and online advertising, more attention to product visuals leads to more favorable product and advertisement attitudes, higher purchase intent, and greater usage expectations (Zhang and Yuan, 2018; Wang et al., 2020; Chen-Sankey et al., 2025).

Text elements provide the advertisement’s semantic foundation by guiding comprehension and clarifying persuasive intent, helping audiences assess risks and benefits (van Reijmersdal et al., 2016; Aikin et al., 2023). Attention to health claims, reviews, and informational text supports more positive product and advertising evaluations and stronger purchase intentions (Chiu and Chang, 2020; Chen et al., 2022; Jin et al., 2023; Chen-Sankey et al., 2024).

Taken together, these findings suggest that visual attention to endorsers, products, and text not only occurs but also drives the evaluative processes underlying persuasion. To capture this, we adopt total fixation duration (TFD)—the cumulative fixation time within an area of interest (AOI)—as the primary index of visual attention, since it reflects sustained processing rather than early orienting (Holmqvist et al., 2011; Duchowski, 2017). TFD is widely used in marketing research to link advertising viewing to subsequent attitudes and behaviors (Pieters and Warlop, 1999; Wedel and Pieters, 2008; van Loon et al., 2022; Aikin et al., 2023). Decision-science studies further show that longer gaze duration both reflects and shapes value computation and choice, offering a mechanistic pathway from attention depth to persuasion (Shimojo et al., 2003; Krajbich et al., 2010; Orquin and Mueller Loose, 2013). Accordingly, we propose the following hypotheses:

*H2a*: Visual attention (TFD) to the endorser, product, and text significantly affects advertising attitude.

*H2b*: Visual attention (TFD) to the endorser, product, and text significantly affects purchase intention.

### 2.3 Influencer type as a moderator of the attention–persuasion link

Posts by human and virtual influencers expose audiences to distinct source cues at the interface level. Drawing on the Computers as Social Actors framework (Reeves and Nass, 1996), viewers often perceive mediated sources as social actors. Consequently, attention operates as an input into interface heuristics, in which emotional and evaluative responses depend on processing fluency—the ease with which a stimulus is cognitively processed (Sundar, 2008; Reber et al., 2004; Carr et al., 2017). High processing fluency elicits more positive evaluations, whereas low fluency induces cognitive conflict and discomfort (Winkielman and Cacioppo, 2001), reflecting differences in perceived familiarity and processing effort (Carr et al., 2017). Thus, attention influences persuasion indirectly through a fluency-based mechanism rather than directly translating into attitude change (Reber et al., 1998; Storme et al., 2015).

In advertising that features human versus virtual influencers, this fluency-based mechanism may be further shaped by the perceived human-likeness of the source. We conceptualize human-likeness along two complementary dimensions: visual appearance and mind perception. The joint configuration of these dimensions determines processing fluency and, ultimately, persuasive effectiveness (Yamada et al., 2013; Park and Sung, 2023; Stein et al., 2024).

Visual human-likeness (appearance-based human-likeness) reduces category ambiguity and mitigates uncanny valley effects (Yamada et al., 2013; Cheetham et al., 2014; Chattopadhyay and MacDorman, 2016), whereas mental human-likeness (mind perception) enhances credibility, empathy, and social acceptance (Waytz et al., 2010; Yam et al., 2021). When both dimensions align to form a fluent, human-typical representation, attention more readily transforms into persuasion (Winkielman et al., 2003; Reber et al., 2004; Alter and Oppenheimer, 2009).

Human influencers provide familiar facial cues, categorical clarity, and authentic experience, facilitating fluent decoding and enhancing credibility heuristics (Lick and Johnson, 2013; Pilgrim and Bohnet-Joschko, 2019; Sokolova and Kefi, 2020). In contrast, virtual endorsements—though visually engaging—can reduce processing fluency when near-human realism feels “not quite right,” prompting heightened vigilance and counterarguing (MacDorman and Chattopadhyay, 2016; MacDorman, 2019). This pattern reflects uncanny valley effects, where imperfect human-likeness reduces trust and comfort (Mori et al., 2012; Song and Shin, 2024; Stein et al., 2024). Accordingly, influencer type moderates the link between visual attention and persuasion by shaping the integrated state of visual and mental human-likeness that governs processing fluency and perceived trustworthiness. We therefore hypothesize:

*H3a*: Influencer type (human vs. virtual) moderates the effect of visual attention (TFD) to the endorser, product, and text on advertising attitude, such that the relationship is stronger for human than for virtual influencers.

*H3b*: Influencer type (human vs. virtual) moderates the effect of visual attention (TFD) to the endorser, product, and text on purchase intention, such that the relationship is stronger for human than for virtual influencers.

## 3 Materials and methods

### 3.1 Participants

Data collection took place between October and November 2023 at a large national university in South Korea. Participants were undergraduate or graduate students currently enrolled and residing in South Korea. Recruitment was conducted via the university online bulletin boards and student organization channels. Eligibility criteria required participants to be at least 19 years old, have normal or corrected-to-normal vision, and be able to attend an on-site eye-tracking session.

An a priori sample size check was conducted in G*Power 3.1 (Faul et al., 2009), assuming a moderate effect size (*d* = 0.50) (Prichard et al., 2023), α = 0.05, and power = 0.80, which indicated a minimum of 103 participants. We recruited above this target to allow for exclusions. Because our focal analyses used hierarchical multiple regressions with interaction (moderation) terms, which generally require larger samples than simple mean comparisons, this calculation is best viewed as a feasibility check for detecting moderate main effects; power for interaction terms is likely limited. In line with this, moderation findings in the Results are interpreted cautiously, with emphasis on standardized coefficients and 95% confidence intervals rather than binary significance tests.

Exclusion criteria were specified a priori and monitored during data collection. Participants were excluded if they failed eye-tracker calibration, had insufficient eye-tracking data quality, or had missing primary survey outcomes. To maintain gender balance across cells, recruitment continued until each experimental cell reached the target of *n* = 30 with a 1:1 female–male quota. In total, 154 East Asian university students were randomized; 13 were excluded for calibration failure, 18 for insufficient data quality, and 3 for missing primary outcomes, yielding a final analytic sample of 120 participants (per cell *n* = 30; 50% female; *M* age = 24.7, *SD* = 3.1, range = 19–35). Exclusion rates did not differ across conditions [χ^2^(3) = 1.88, *p* = 0.598; see Supplementary Tables 1, 2]. Descriptive characteristics of the sample (age, gender, education level, household income, Instagram use) are presented in Table 1.

**TABLE 1:** Demographic characteristics of participants.

Demographic characteristic	Full sample (*N* = 120), n (%)
**Age group, n (%)**	
19–21	16 (13.3%)
22–24	46 (38.3%)
25–27	32 (26.7%)
≥ 28	26 (21.7%)
**Gender, n (%)**	
Female	60 (50.0%)
Male	60 (50.0%)
**Educational level, n (%)**	
Lower than bachelor’s degree	60 (50.0%)
Bachelor’s degree	37 (30.8%)
Higher than bachelor’s degree	23 (19.2%)
**Household income, USD/year, n (%)**	
<23,000	26 (21.7%)
23,000–38,460	60 (50.0%)
More than 38,460	34 (28.3%)
**Instagram use, hours/day, n (%)**	
0–0.5 h	74 (61.7%)
0.5–1 h	32 (26.7%)
1–1.5 h	11 (9.5%)
> 1.5 h	3 (2.5%)

### 3.2 Ethics statement

This study received approval from the Institutional Review Board of Jeonbuk National University (IRB No. JBNU2023-09-031-005). All procedures were conducted in accordance with the Declaration of Helsinki. Written informed consent was obtained from all participants, who retained the right to withdraw at any time without penalty. Participants received cultural goods vouchers as compensation.

### 3.3 Manipulation check

To help isolate the effects of influencer type (human vs. virtual), we used a multi-stage selection process aimed at comparability on three key perceptual attributes: familiarity, attractiveness, and favorability, which influence message reception in social media (Myers, 2021; Iqbal et al., 2023; Kim and Park, 2023).

We first assembled an initial pool of eight macro influencers: four human (H1–H4) and four virtual (V1–V4). Each had more than 100,000 Instagram followers. To minimize prior familiarity and cultural in-group biases, we purposively sampled Western influencers expected to have low name recognition among East Asian young adults. We then conducted a pretest in which ten university students (60% female; *M* age = 21.9, *SD* = 2.12) evaluated each influencer’s profile—one profile photo, three posts, and bio—on three focal traits using 7-point Likert scales.

Friedman tests across the four accounts within each type were used to assess within-type differences. Human influencers showed no within-type differences in familiarity, attractiveness, or favorability [χ^2^(3) = 0.500–1.933, *p* ≥ 0.586]. Virtual influencers differed in familiarity [χ^2^(3) = 18.280, *p* < 0.001; see Supplementary Tables 3, 4]. We therefore removed the virtual account with atypically high familiarity; follow-up testing in this small pretest sample did not detect any remaining differences among the virtual accounts. From this set, we retained two per type (H1, H2; V2, V3) whose mean trait profiles were most similar. Within this small pretest sample (*n* = 10), paired Wilcoxon signed-rank tests on participant-level means did not detect statistically significant human–virtual differences in any of the three attributes (*p* ≥ 0.332; see Supplementary Table 5). Overall, these procedures yielded stimulus sets that were approximately comparable across influencer types. Given the relatively small pretest sample, however, these results should be interpreted as providing an approximate rather than formal test of equivalence.

### 3.4 Experimental stimuli and research design

We standardized advertisement images and copy in Photoshop to control visual variables. Core elements (face, product, and text) were scaled according to strict proportional rules so that their on-screen areas were approximately equal across stimuli, and identical advertising copy was used in all conditions. For the stimuli, we selected a vitamin C dietary supplement specifically designed for adults in their 20s and 30s. Because vitamin C supplements have few gender associations, this choice helped to better isolate influencer effects. To minimize prior brand familiarity, we developed a fictitious brand (UISICARI). To control for gender influences on purchase intentions (Beldad et al., 2016), each influencer type—human and virtual—was represented by one male and one female endorser, both of whom were shown actively promoting the product.

The study employed a between-subjects 2 × 2 factorial design—Influencer type (human vs. virtual) × Endorser gender (male vs. female)—with 30 participants per cell (A1: human male; A2: human female; B1: virtual male; B2: virtual female), randomly assigned. Each participant was exposed only to the stimuli of their assigned cell: Two advertisement images featuring the same endorser, created in a uniform visual style. After viewing the two images from their assigned cell, participants completed one unified survey assessing advertising effectiveness.

### 3.5 Experimental procedure

Participants were seated 65 cm from a 24-inch monitor (1,920 × 1,080, 60 Hz) in a quiet, windowless room with neutral gray walls and no screen glare. After posture stabilization and instructions to minimize head movement, a 9-point calibration was performed using the Tobii Pro Nano (60 Hz) with validation criteria of mean error < 0.5° and maximum error < 1.0°.

**FIGURE 1:**
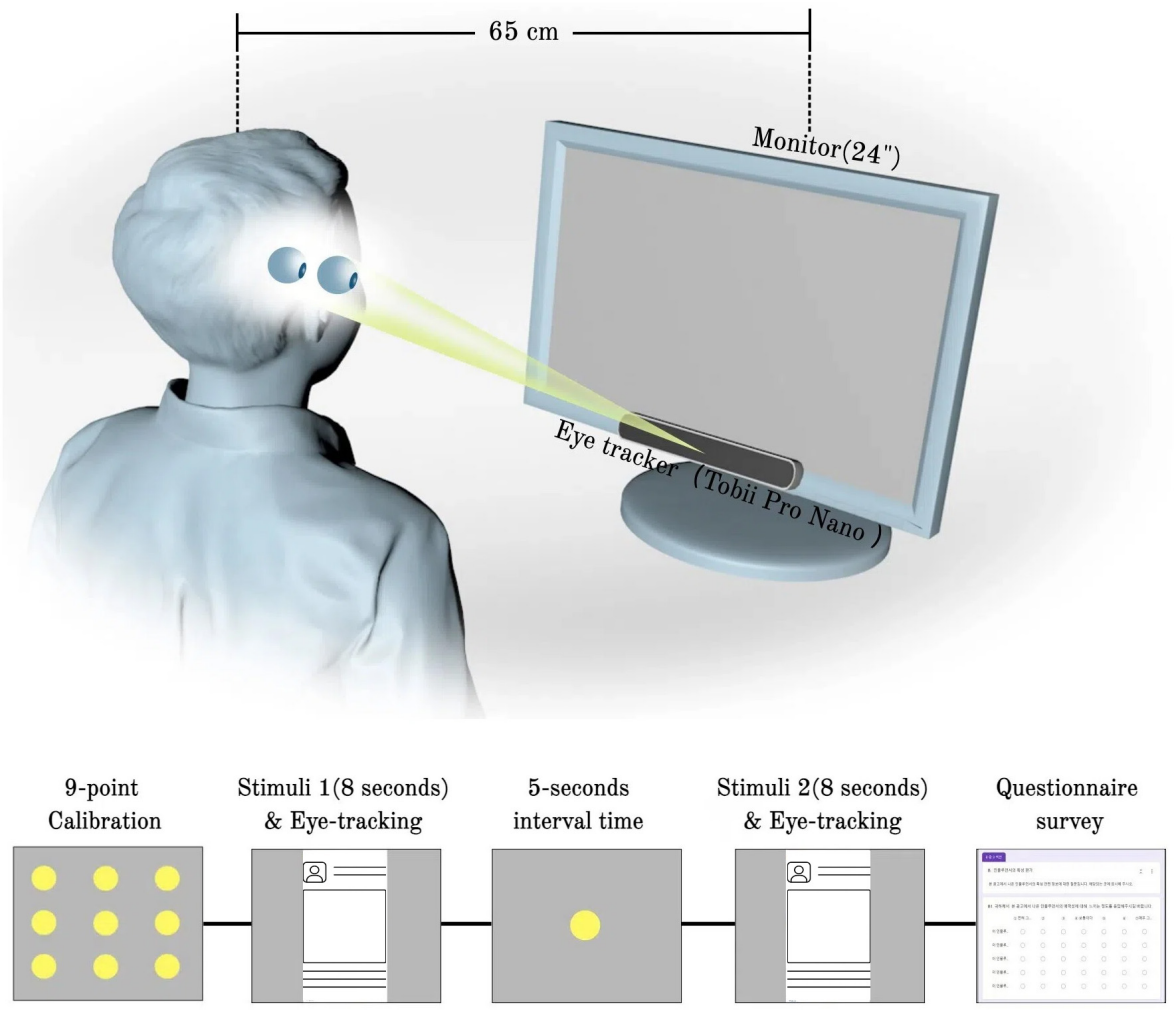
Procedure of eye-tracking experiment.

Two advertisement images were shown sequentially in full-screen mode, each for 8 s, with a 5-s gray inter-stimulus interval. Presentation order was counterbalanced across participants. The 8-s duration aligns with established eye-tracking protocols, which show stable attention allocation for static advertisements (Radach et al., 2003; Blanc and Brigaud, 2014; Brigaud et al., 2021). Immediately after exposure, participants completed a questionnaire that assessed demographics, social media usage, advertising attitudes, and purchase intentions (see Figure 1 for a schematic of the procedure).

Gaze data were processed in Tobii Pro Lab using the I-VT filter with 60 Hz parameters. Settings included a velocity threshold of 30°/s, minimum fixation duration of 60 ms, fixation merging for separations < 75 ms and < 0.5° visual angle, and a 20 ms velocity window. Missing segments < 75 ms were linearly interpolated; longer gaps were treated as blinks and excluded. Trials with valid-sample ratios < 80% or fewer than three remaining fixations after filtering were deemed invalid and excluded.

### 3.6 Measures

#### 3.6.1 Covariates

To examine the relationship between eye movements and advertising effectiveness, we included participants’ demographic characteristics (gender and age) and recognition accuracy (human vs. virtual influencer) as covariates. The final sample comprised 120 participants (60 males, 60 females; *M* age = 24.7 years). Recognition accuracy was measured with a forced-choice check in which participants classified the influencer as “human,” “virtual,” or “uncertain;” “uncertain” responses were coded as incorrect. In the human condition, 71.7% of participants correctly identified the influencer as human, while in the virtual condition 81.7% correctly identified the influencer as virtual. Further details are reported in Supplementary Table 6.

#### 3.6.2 Dependent variables

The advertisement’s effectiveness was measured on two main dimensions: attitude toward the advertisement and purchase intention. Attitude was assessed using five 7-point semantic differential items adapted from MacKenzie and Lutz (1989) and Dimofte et al. (2015). The five items were averaged to create a composite score, which demonstrated strong internal consistency (Cronbach’s α = 0.92, *M* = 3.66, *SD* = 0.98).

Purchase intention was assessed using three analogous 7-point semantic differential items, based on Dodds et al. (1991), capturing participants’ likelihood of buying the advertised product. This index also showed high reliability (Cronbach’s α = 0.89, *M* = 2.95, *SD* = 0.87). Full item wording, response scales, anchor labels, scoring rules, and aggregation procedures for all measures are reported in Supplementary Table 7.

#### 3.6.3 Eye-tracking variables

To capture the distribution of attention among the core advertisement elements, we defined three AOIs: the endorser’s face, the product, and the text. The endorser’s face AOI was an oval extending from the forehead to the chin, including the zygomatic regions. The product AOI was defined as a rectangular region enclosing the product, whereas the text AOI was defined as a rectangular region encompassing the advertisement copy and the influencer/account handle (see Supplementary Figure 1). All AOIs were drawn by a trained research assistant following a standardized protocol developed by the research team and checked by the first author.

Because each participant viewed two images, TFDs for each AOI were averaged within participant across the two images to produce a single mean TFD per participant. As AOIs differed in on-screen size—potentially affecting observed attention—we calculated area-normalized TFD for each AOI by dividing TFD by the AOI area (in pixels). Additional details on AOI pixel sizes and visual salience indicators are provided in Supplementary Table 8.

### 3.7 Analysis plan

All analyses were conducted using IBM SPSS Statistics 27.0 (IBM Corp.). All tests were two-tailed, with an α level of 0.05. Hierarchical multiple regression models were estimated separately for advertising attitude and purchase intention.

Before conducting the primary analyses, we examined descriptive statistics and bivariate relationships for the key variables (see Supplementary Tables 9, 10). We performed a two-step hierarchical regression analysis. In Step 1, we included covariates (age, gender, and influencer recognition accuracy), the main predictor (influencer type), and three eye-tracking indicators: TFD on the endorser’s face, the product, and the text. Step 2 incorporated moderation by adding interaction terms between influencer type and TFD for each element. All predictors involved in interactions were mean-centered. To ensure robustness, multicollinearity was assessed using variance inflation factors (VIFs), all of which were below 3.3. Residuals were visually inspected for normality, with no significant deviations observed.

## 4 Results

### 4.1 Effect of influencer type on advertising effectiveness

Patterns in Tables 2 and 3 are consistent with influencer type being associated with both advertising attitude and purchase intention. Influencer type was dummy-coded (0 = human, 1 = virtual); so negative coefficients indicate higher scores for the human condition. In Model 1, human influencers were associated with more positive attitudes [*b* = −0.644, *p* < 0.05, 95% CI (−1.052, −0.236)] and higher purchase intentions [*b* = −0.696, *p* < 0.001, 95% CI (−1.043, −0.350)] than virtual influencers. Model 2 similarly showed that human influencers were inked with more positive advertising attitudes [*b* = −0.673, *p* < 0.01, 95% CI (−1.063, −0.282)] and higher purchase intentions [*b* = −0.697, *p* < 0.001, 95% CI (−1.034, −0.282)]. These findings are consistent with H1a and H1b.

**TABLE 2:** The impact of influencer type and visual attention on advertising attitude.

Variables	Advertising attitude (Model 1)						Advertising attitude (Model 2)					
	(*N* = 120)						(*N* = 120)					
	*b*	SE	*t*	*p*	95% CI		*b*	SE	*t*	*p*	95% CI	
					Lower	Upper					Lower	Upper
**Demographics**												
Age	0.010	0.030	0.326	0.745	−0.049	0.068	0.020	0.026	0.772	0.442	−0.032	0.072
Gender	−0.025	0.177	−0.142	0.887	−0.375	0.325	−0.041	0.159	−0.257	0.798	−0.356	0.274
**Influencer recognition**												
Recognition accuracy^a^	0.073	0.223	0.329	0.743	−0.368	0.515	0.132	0.195	0.677	0.500	−0.255	0.520
**Influencer feature**												
Influencer type^b^	−0.644	0.206	−3.130	0.002	−1.052	−0.236	−0.673	0.197	−3.415	0.001	−1.063	−0.282
**Visual attention^c^**												
Endorser TFD	0.107	0.044	2.441	0.016	0.020	0.193	0.200	0.055	3.620	< 0.001	0.091	0.310
Product TFD	0.036	0.026	1.390	0.167	−0.015	0.088	0.022	0.024	0.910	0.365	−0.026	0.069
Text TFD	0.151	0.058	2.621	0.010	0.037	0.265	−0.012	0.070	−0.177	0.860	−0.152	0.127
**Interactions**												
Influencer type × endorser TFD							−0.536	0.111	−4.844	< 0.001	−0.756	−0.317
Influencer type × product TFD							0.050	0.047	1.049	0.297	−0.044	0.144
Influencer type × text TFD							0.083	0.143	0.583	0.561	−0.200	0.366
*R* ^2^	0.116						0.342					
Δ*R*^2^	–						0.226					
*F*	*F*(7, 112) = 2.103, *p* = 0.049						*F*(10, 109) = 5.672, *p* < 0.001					

*^a^*Recognition accuracy: 0 = incorrect identification; 1 = correct identification;

*^b^*Influencer type: 0 = human influencer; 1 = virtual influencer;

*^c^*TFD values were area-normalized by dividing the TFD by the size of each AOI. Model 1: The association of advertising attitude (the dependent variable) with demographics (age and gender), influencer type, recognition accuracy, and visual attention (endorser TFD, product TFD, and text TFD). Model 2: Model 1 + terms of interaction (influencer type × endorser TFD, influencer type × product TFD, and influencer type × text TFD).

### 4.2 Effect of visual attention on advertising effectiveness

Visual attention to advertisement components was associated with both advertising effectiveness. In the main-effects model, greater attention to the endorser [*b* = 0.107, *p* < 0.05, 95% CI (0.020, 0.193)] and to the text [*b* = 0.151, *p* < 0.05, 95% CI (0.037, 0.265)] were positively related to advertising attitude (Table 2, Model 1). Attention to the product, however, was not a significant predictor [*b* = 0.036, *p* > 0.05, 95% CI (−0.015, 0.088)]. These results provide partial and tentative support for H2a.

With regard to purchase intention, both endorser attention [*b* = 0.124, *p* < 0.01, 95% CI (0.050, 0.198)] and text attention [*b* = 0.168, *p* < 0.01, 95% CI (0.071, 0.265)] were positively associated with purchase intention, whereas product attention was again not significant [*b* = 0.035, *p* > 0.05, 95% CI (−0.009, 0.079)] (Table 3, Model 1). These patterns offer partial evidence for H2b.

**TABLE 3:** The impact of influencer type and visual attention on purchase intention.

Variables	Purchase intention (Model 1)						Purchase intention (Model 2)					
	(*N* = 120)						(*N* = 120)					
	*b*	SE	*t*	*p*	95% CI		*b*	SE	*t*	*p*	95% CI	
					Lower	Upper					Lower	Upper
**Demographics**												
Age	−0.010	0.025	−0.398	0.691	−0.060	0.040	−0.001	0.023	−0.053	0.958	−0.046	0.043
Gender	−0.051	0.150	−0.339	0.735	−0.349	0.247	−0.048	0.137	−0.352	0.726	−0.321	0.224
**Influencer recognition**												
Recognition accuracy^a^	0.037	0.190	0.194	0.847	−0.339	0.412	0.083	0.169	0.493	0.623	−0.252	0.418
**Influencer feature**												
Influencer type^b^	−0.696	0.175	−3.978	< 0.001	−1.043	−0.350	−0.697	0.170	−4.089	< 0.001	−1.034	−0.359
**Visual attention^c^**												
Endorser TFD	0.124	0.037	3.34	0.001	0.050	0.198	0.194	0.048	4.052	< 0.001	0.099	0.289
Product TFD	0.035	0.022	1.577	0.118	−0.009	0.079	0.025	0.021	1.223	0.224	−0.016	0.066
Text TFD	0.168	0.049	3.438	0.001	0.071	0.265	0.026	0.061	0.424	0.672	−0.095	0.146
**Interactions**												
Influencer type × endorser TFD							−0.453	0.096	−4.735	< 0.001	−0.643	−0.264
Influencer type × product TFD							0.012	0.041	0.300	0.764	−0.069	0.094
Influencer type × text TFD							0.032	0.123	0.258	0.797	−0.213	0.276
*R* ^2^	0.188						0.375					
Δ*R*^2^	–						0.187					
*F*	*F*(7, 112) = 3.696, *p* < 0.001						*F*(10, 109) = 6.533, *p* < 0.001					

*^a^*Recognition accuracy: 0 = incorrect identification; 1 = correct identification;

*^b^*Influencer type: 0 = human influencer; 1 = virtual influencer;

*^c^*TFD values were area-normalized by dividing the TFD by the size of each AOI. Model 1: The association of purchase intention (the dependent variable) with demographics (age and gender), influencer type, recognition accuracy, and visual attention (endorser TFD, product TFD, and text TFD). Model 2: Model 1 + terms of interaction (influencer type × endorser TFD, influencer type × product TFD, and influencer type × text TFD).

### 4.3 Moderation by influencer type

To examine H3a and H3b, we investigated whether influencer type moderated the effect of endorser TFD on advertising attitude and purchase intention. As shown in Table 2 (Model 2) and Table 3 (Model 2), the interaction between endorser TFD and influencer type showed a pattern consistent with a possible moderating effect on both advertising attitude [*b* = −0.536, *p* < 0.001, 95% CI (−0.756, −0.317)] and purchase intention [*b* = −0.453, *p* < 0.001, 95% CI (−0.643, −0.264)]. By contrast, the interaction patterns involving product TFD and text TFD did not show clear trends. While the observed trends resemble the relationships proposed in H3a and H3b, they remain exploratory given the limited sample size.

**FIGURE 2:**
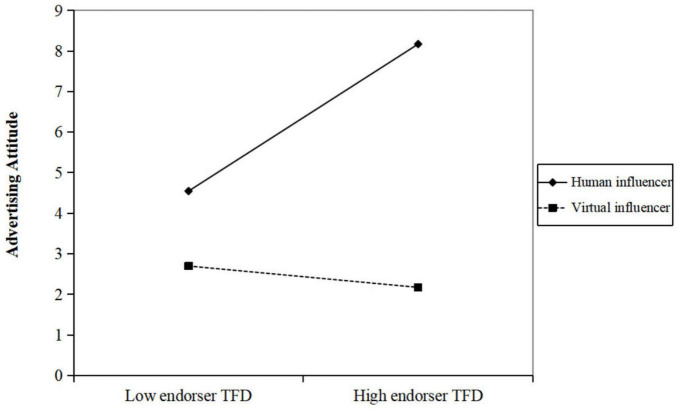
The interaction effect of influencer type and endorser TFD on advertising attitude.

**FIGURE 3:**
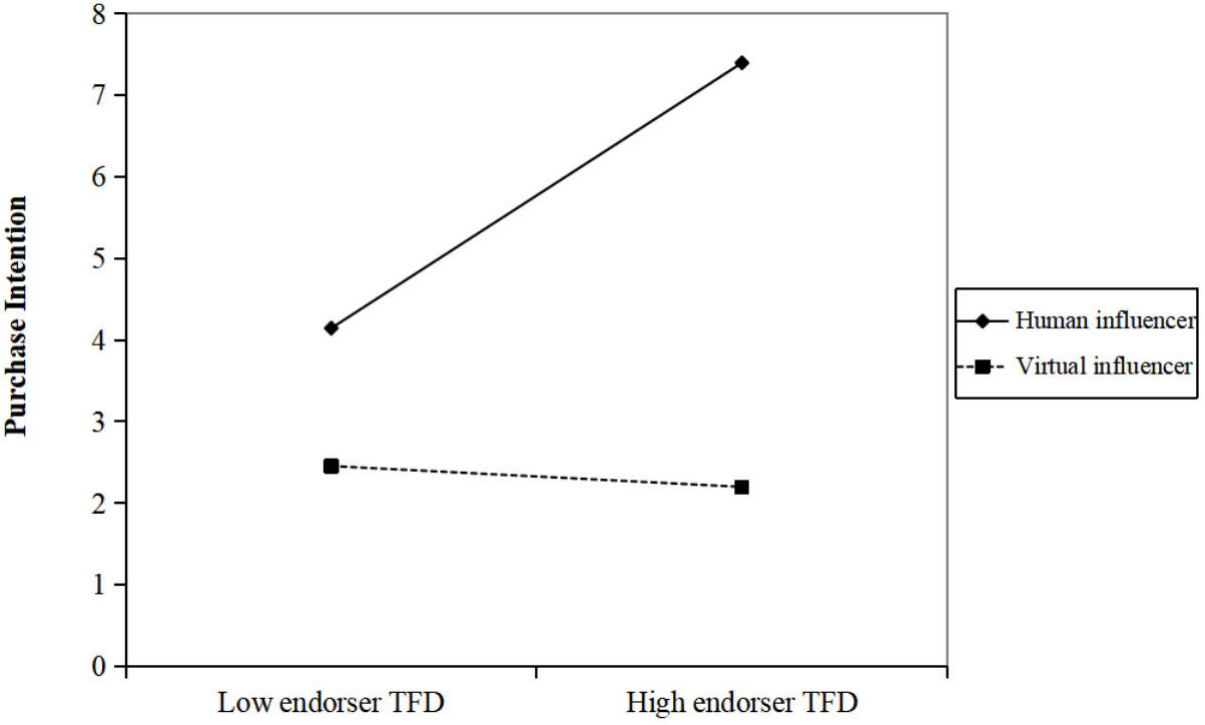
The interaction effect of influencer type and endorser TFD on purchase intention.

Simple-slope analyses indicated that, in the human influencer condition, greater endorser TFD was associated with more positive attitudes [*b*_simpl*e*_ = 0.468, *p* < 0.001, 95% CI (0.326, 0.611)], whereas in the virtual influencer condition the association was not evident [*b*_simpl*e*_ = −0.068, *p* > 0.05, 95% CI (−0.235, 0.099)] (Figure 2). Similarly, for purchase intention, greater TFD was associated with higher intention in the human condition [*b*_simpl*e*_ = 0.420, *p* < 0.001, 95% CI (0.297, 0.543)], while no clear association was observed in the virtual condition [*b*_simpl*e*_ = −0.033, *p* > 0.05, 95% CI (−0.177, 0.111)] (Figure 3). These patterns tentatively suggest that the relationship between fixation duration and advertising outcomes may be stronger for human endorsers than for virtual endorsers.

### 4.4 Differences in visual attention by influencer type

Descriptive analyses showed clear differences in visual attention based on influencer type (Table 4). Participants spent more time looking at the virtual influencer’s face (*M* = 5.707, *SD* = 2.836) than at the human influencer’s face (*M* = 4.255, *SD* = 1.593), *t* = −3.457, *p* < 0.01, 95% CI [−2.286, −0.618]. Participants also focused more on the product in advertising featuring human influencers (*M* = 7.142, *SD* = 3.237) than in advertising featuring virtual influencers (*M* = 4.971, *SD* = 4.169), *t* = 4.227, *p* < 0.01, 95% CI [0.822, 3.521]. Participants paid more attention to text in virtual influencer conditions (*M* = 3.243, *SD* = 2.050) than in human influencer conditions (*M* = 2.301, *SD* = 1.813), *t* = −2.666, *p* < 0.01, 95% CI [−1.642, −0.242].

**TABLE 4:** Differences in visual attention according to influencer types.

Variables	Group (human *n* = 60, virtual *n* = 60)	*M (SD)*		*t*	*p*	95% CI	
		Group	Total			Lower	Upper
Endorser TFD	Human influencer	4.255 (1.593)	4.981 (2.403)	−3.457	0.001	−2.286	−0.618
	Virtual influencer	5.707 (2.836)					
Product TFD	Human Influencer	7.142 (3.237)	6.056 (3.873)	4.227	0.002	0.822	3.521
	Virtual Influencer	4.971 (4.169)					
Text TFD	Human Influencer	2.301 (1.813)	2.772 (1.984)	−2.666	0.009	−1.642	−0.242
	Virtual Influencer	3.243 (2.050)					

TFD values were area-normalized by dividing the TFD by the size of each AOI.

## 5 Discussion

### 5.1 Key findings

The findings highlight persuasive differences between human and virtual influencers in shaping young adults’ responses to health supplement advertising. Compared with human endorsers, advertisements featuring virtual influencers tended to be associated with lower advertising attitude and weaker purchase intention. This pattern is tentatively consistent with recent work on virtual influencer effects (Franke et al., 2023; Lou et al., 2023; Barari et al., 2025). Endorsements perceived as genuine or transparent tend to elicit more favorable advertising evaluation and stronger purchase intention (Audrezet et al., 2020; Hasan et al., 2024; Liu and Zheng, 2024). This effect is especially important for credence goods such as health supplements, whose quality cannot be easily assessed by consumers; in such cases, audiences depend on credible human cues to reduce uncertainty (Dulleck and Kerschbamer, 2006).

Visual attention patterns also differed by influencer type. Virtual influencers drew more gaze to facial and textual elements, while human influencers demonstrated a stronger ability to direct consumers’ attention toward the product itself. This pattern reflects increased source-checking for nonhuman agents, as viewers seek to identify who is speaking and what is being said near the human–nonhuman boundary. In contrast, human endorsers elicit joint attention, where observers naturally follow the endorser’s gaze or posture toward the referenced object, increasing attention to the product (Friesen and Kingstone, 1998; Lu and van Zoest, 2023). These attentional dynamics tentatively indicate that an endorser’s ontological status redistributes visual attention, reflecting distinct pathways of visual—cognitive processing.

This redistribution of attention may have implications for advertising effectiveness. In the main-effects model, text attention emerged as the strongest predictor, consistent with the view that credence goods often rely on diagnostic verbal claims (Pieters and Wedel, 2004; Dulleck and Kerschbamer, 2006; Cuffaro and Di Giacinto, 2015). When interaction terms were included, the pattern suggested that attention to faces—particularly in relation to influencer type—was differentially associated with outcomes: greater fixation on the endorser’s face was linked to more favorable responses in the human influencer condition but not in the virtual influencer condition. These results support the view that attention functions as input to interface heuristics, whose impact depends on human-likeness processing fluency (Winkielman and Cacioppo, 2001; Sundar, 2008; Carr et al., 2017). Human endorsers’ facial and verbal cues may facilitate perceptions of authenticity, empathy, and categorical clarity that support credibility and persuasion (Reber et al., 2004; Carr et al., 2017; Sokolova and Kefi, 2020; Um, 2022). By contrast, hybrid human–nonhuman cues associated with virtual influencers may reduce processing fluency and increase vigilant evaluation, thereby weakening immediate message acceptance (Alter and Oppenheimer, 2009; MacDorman, 2019). Overall, while both influencer types attract attention, these patterns tentatively suggest that attention is more likely to translate into positive evaluation and purchase intention for human endorsers in our sample.

### 5.2 Theoretical implications

This study offers several theoretical contributions to neuromarketing, influencer marketing, and health communication. First, the findings may help to extend source credibility theory by suggesting a boundary condition in credence-goods contexts. Consistent with prior work emphasizing the centrality of credibility cues (Ohanian, 1990; Dulleck and Kerschbamer, 2006), human endorsers were associated with more favorable persuasive outcomes than computer-generated agents, plausibly due to the authenticity cues they convey as proxies for otherwise unverifiable product quality (Sbaffi and Rowley, 2017; Audrezet et al., 2020).

Second, the present findings may help to illuminate when visual attention is more likely to translate into persuasive outcomes by suggesting a potential underlying mechanism. The observed patterns appear consistent with the notion of human-likeness processing fluency, whereby attention may be more effectively linked with persuasion when endorser cues display human-typical features (Mori et al., 2012; Diel and MacDorman, 2021), are perceived as mentally human-like—showing both agency and experience (Waytz et al., 2010; Gray and Wegner, 2012; Stein et al., 2024)—and are presented in a way that facilitates smooth cognitive processing (Winkielman et al., 2003; Reber et al., 2004; Alter and Oppenheimer, 2009). Departing from existing comparative research on virtual and human endorsers (Lou et al., 2023; Belanche et al., 2024; Deng et al., 2024; Dondapati and Dehury, 2024), the present study tentatively suggests that the link between visual attention, belief formation, and behavioral intention may depend on specific contextual factors, including the processing fluency of human-likeness.

Third, this study demonstrates the value of eye-tracking in capturing preconscious attention. It reveals mechanism-level distinctions in the attention–evaluation process between human and virtual endorsers. Whereas much of the literature has relied on self-reported attitudes and intentions, gaze metrics provide complementary and objective predictors of advertising effectiveness, allowing researchers to probe attention-driven persuasion mechanisms more deeply.

### 5.3 Practical implications

These findings may offer preliminary implications for practitioners in health supplement marketing. The observed pattern suggests that when product quality is difficult to verify, human endorsers may be better positioned to foster more stronger advertising effectiveness, possibly due to the credibility and perceived social connection they convey (Petty and Cacioppo, 1986; Ohanian, 1990; Dulleck and Kerschbamer, 2006; Sokolova and Kefi, 2020). This implies that brands might prioritize human influencers in campaigns where trust and short-term persuasion are central goals. However, because these results come from a single lab-based experiment with student participants in one product category, such recommendations should be considered tentative.

From a design perspective, enhancing processing fluency may facilitate more favorable audience responses. This can be achieved by presenting information in formats that audiences can easily interpret, maintaining a stable visual style, and clearly linking each claim to its supporting evidence. For virtual influencers, it may be beneficial to reduce category ambiguity and minimize vigilance-inducing cues by keeping facial rendering, voice, and motion relatively consistent across touchpoints. Taken together, these practices can increase evaluative ease and reduce cognitive friction (Reber et al., 2004; Alter and Oppenheimer, 2009).

### 5.4 Limitations

Several limitations should be acknowledged. First, stimuli were matched using a brief pretest (*n* = 10), so comparability should be viewed as approximate. We did not measure participants’ prior exposure to the selected influencers, relying instead on the pretest and purposive selection of low-recognition Western accounts. Consequently, familiarity may have varied across participants and could have contributed to differences in both visual attention and evaluative outcomes.

Second, constructs such as authenticity, human-likeness, and processing fluency were invoked to interpret observed gaze patterns and persuasive outcomes. These should be treated as inferred mechanisms rather than directly measured variables, so interpretations regarding their role in attention and persuasion should be considered with caution.

Third, our sample comprised Korean university students. Therefore, the findings are restricted to East Asian student populations and may not generalize to more individualistic cultures, older adults, or nonstudent groups. Cross-cultural and broader demographic replication is warranted.

Fourth, AOIs were defined by a single trained coder following a detailed protocol and verified by the first author. Because only one coder was involved, inter-coder reliability could not be calculated. Future eye-tracking studies using complex advertising stimuli should involve multiple independent coders to enhance the robustness of gaze-based inferences.

Fifth, AOI specification and visual salience may systematically shape visual attention. Differences in AOI definition, as well as basic visual properties such as placement, contrast, and brightness, can guide gaze allocation. Future research should more explicitly incorporate these visual factors to better disentangle psychological effects from stimulus-driven variation.

Finally, as an exploratory study, we did not preregister AOIs or analyses. Future confirmatory work would benefit from preregistered AOI definitions, exclusion criteria, and analysis plans to further enhance transparency.

### 5.5 Future research directions

In light of these limitations, several avenues for future research emerge. First, future studies could test and refine the proposed pathway by incorporating brief, reliable measures of authenticity, human-likeness, and processing fluency alongside eye-tracking data. This would enable testing mediation models linking attention to attitudes and intentions, clarifying when attention translates into persuasion and identifying key factors that enhance persuasive effectiveness.

Second, future research should test these mechanisms across health product categories that vary in risk and involvement to establish boundary conditions and inform policy. For example, studies should examine high-risk claims (e.g., disease prevention or therapeutic assertions) and measure perceived medical legitimacy and safety to assess how risk and credibility shape persuasion.

Third, future eye-tracking work should examine when viewers first fixate and how their gaze shifts across elements. Assessing the sequence of attention—for instance, from face to text to product—and its relationship with downstream outcomes (e.g., click-through or purchase intention) would extend prior work on attention and conversion (Pieters and Wedel, 2004; Wedel and Pieters, 2008; Teixeira et al., 2012). Experimental designs could manipulate gaze direction, pointing cues, or copy placement to test whether optimized attention flows can mitigate virtual influencers’ authenticity gap and improve the effectiveness of health-related influencer advertising.

## 6 Conclusion

In health supplement advertising, the type of endorser appears to influence how visual attention may translate into persuasion-related outcomes. Eye-tracking results suggest that virtual influencers tend to attract greater gaze allocation toward faces and text, yet this increased attention may not consistently correspond with favorable attitudes or behavioral intentions. In contrast, human endorsers are associated with comparatively stronger evaluations and higher purchase intentions. These patterns imply that the persuasive role of visual attention could be shaped by the perceived fluency of processing human-likeness. When visual human-likeness (appearance-based human-likeness) and mental human-likeness (mind perception of agency and experience) are processed more fluently, attention may more readily contribute to positive evaluations (Winkielman et al., 2003; Reber et al., 2004; Alter and Oppenheimer, 2009; Waytz et al., 2010; Gray and Wegner, 2012; Mori et al., 2012; Diel and MacDorman, 2021; Stein et al., 2024).

Theoretically, this study may help shift the focus from simply identifying “which type performs better” toward understanding when and why attentional processes become persuasive. It also helps to specify mechanism-level and category-sensitive boundary conditions that can clarify how attention operates across varying degrees of human-likeness. Methodologically, the results highlight the potential value of eye-tracking as a complement to self-report measures, enabling detection of preconscious processes that may shape persuasive outcomes. Managerially, the patterns observed suggest a preliminary hypothesis: human endorsers may be more promising for claims that heavily depend on credibility. Overall, this exploratory experiment offers a tentative, testable framework for how attention may be converted into persuasion and provides a roadmap for future research to evaluate targeted interventions.

## Data Availability

The raw data supporting the conclusions of this article will be made available by the authors, upon request. Requests to access these datasets should be directed to Mengqi Du, dumengqi08@gmail.com.
