# Supplementary material for: Persuasive differences between human and virtual influencers in health supplement advertising: evidence from eye-tracking
Source: Front Psychol. 2026 Jan 6;16:1692737. doi: 10.3389/fpsyg.2025.1692737 (PMC12816186; doi:10.3389/fpsyg.2025.1692737)
Supplement: Supplementary file 1 [file Supplementary_file_1.docx]

Supplementary Materials

**TABLE S1** Condition-specific exclusions after assignment.

| Condition | Assigned (*n*) | Excluded (*n*) | Retained (*n*) | Exclusion rate (%) |
| --- | --- | --- | --- | --- |
| A | 42 | 12 | 30 | 28.6 |
| B | 37 | 7 | 30 | 18.9 |
| C | 39 | 9 | 30 | 23.1 |
| D | 36 | 6 | 30 | 16.6 |
| Total | 154 | 34 | 120 | 22.1 |

Notes. “Assigned” includes all randomized participants per condition (final analyzed + excluded).

Rates are per condition; percentages may not sum exactly due to rounding.

**TABLE S2** Test of differential exclusion by condition.

| Test | *χ²* | *df* | *N* | *p* | Cramér’s V |
| --- | --- | --- | --- | --- | --- |
| Pearson’s chi-square | 1.88 | 3 | 154 | 0.598 | 0.11 |

**TABLE S3** Friedman tests across four influencers within type (human influencers).

| Attributes | H1, *M (SD)* | H2, *M (SD)* | H3, *M (SD)* | H4, *M (SD)* | *χ^2^* | *p* |
| --- | --- | --- | --- | --- | --- | --- |
| Familiarity | 3.800 (0.919) | 3.900 (0.876) | 4.100 (1.197) | 3.900 (1.101) | 0.500 | 0.919 |
| Attractiveness | 5.200 (1.135) | 4.800 (0.632) | 4.800 (0.632) | 4.900 (0.316) | 0.750 | 0.861 |
| Favorability | 4.600 (0.843) | 5.000 (1.333) | 5.000 (1.054) | 5.200 (0.919) | 1.933 | 0.586 |

Note. *N* = 10; H = human influencers; *M (SD)* = Mean ± (standard).

**TABLE S4** Friedman tests across four influencers within type (virtual influencers).

| Attributes | V1, *M (SD)* | V2, *M (SD)* | V3, *M (SD)* | V4, *M (SD)* | *χ^2^* | *p* |
| --- | --- | --- | --- | --- | --- | --- |
| Familiarity | 2.500 (0.850) | 3.600 (1.075) | 4.300 (0.675) | 4.400 (0.966) | 18.280 | < 0.001 |
| Attractiveness | 5.600 (0.966) | 5.200 (1.135) | 5.200 (1.033) | 5.000 (1.054) | 2.362 | 0.501 |
| Favorability | 4.800 (1.033) | 5.100 (1.287) | 5.300 (0.949) | 5.100 (0.994) | 2.962 | 0.398 |

Note. *N* = 10; V = virtual influencers; *M (SD)* = Mean (standard).

**TABLE S5** Wilcoxon signed-rank tests across two types of influencers.

| Attributes | Type | *M (SD)* | *Mdn* |
| --- | --- | --- | --- |
| Familiarity | Human | 3.850 (0.709) | 4.000 |
|  | Virtual | 3.950 (0.762) | 4.000 |
|  | Wilcoxon: *W* = 12.000, *p* = 0.730, *r* = 0.001 | | |
| Attractiveness | Human | 5.000 (0.577) | 5.000 |
|  | Virtual | 5.200 (0.715) | 5.500 |
|  | Wilcoxon: *W* = 15.500, *p* = 0.392, *r* = 0.271 | | |
| Favorability | Human | 5.050 (0.497) | 5.000 |
|  | Virtual | 5.200 (0.789) | 5.000 |
|  | Wilcoxon: *W* = 6.000, *p* = 0.332, *r* = 0.307 | | |

Note. *N* = 10; *M (SD)* = Mean (standard); Values are participant-level means across two human (H1, H2) and two virtual (V2,V3) influencers per trait.

**TABLE S6** Confusion matrix for influencer recognition.

| Condition | Recognized as human  n (%)^a^ | Recognized as virtual  n (%)^a^ | Uncertain  n (%)^a^ | Total  n (%)^a^ | Recognition accuracy  n (%)^a^ |
| --- | --- | --- | --- | --- | --- |
| Human influencer | 43 (71.7%) | 5 (8.3%) | 12 (20.0%) | 60 (50.0%) | 43 (71.7%) |
| Virtual influencer | 7 (11.7%) | 49 (81.7%) | 4 (6.7%) | 60 (50.0%) | 49 (81.7%) |
| Total | 50 (41.7%) | 54 (45.0%) | 16 (13.3%) | 120 (100.0%) | − |

Note. ^a^Data are presented as number (%); Recognition accuracy treating “Uncertain” as incorrect.

**TABLE S7** Standardized factor loadings and scale reliability for study variables.

| Variables (sources) | Items | Factor loadings | Cronbach’s *α* |
| --- | --- | --- | --- |
| Advertising attitude  (MacKenzie and Lutz, 1989; Dimofte et al., 2015). | This advertisement is bad–good. | 0.860 | 0.916 |
|  | This advertisement is disliked–liked. | 0.865 |  |
|  | This advertisement is unfavorable–favorable. | 0.824 |  |
|  | This advertisement is unpleasant–pleasant. | 0.904 |  |
|  | This advertisement is uninformative–informative. | 0.874 |  |
| Purchase intention (Dodds et al., 1991) | I intend to purchase this product. | 0.919 | 0.886 |
|  | I would consider buying this product. | 0.900 |  |
|  | I will try to buy this product. | 0.891 |  |

Note. 7-point semantic differential; anchors arranged so that 7 = more positive/ stronger intention. Items averaged to form the composite; no reverse-coding required. When each variable was assessed, cases with missing data were excluded from analysis.

**TABLE S8** AOI visual salience metrics and pixel sizes across influencer conditions.

| Type | AOI | Mean luminance | RMS contrast | Area pixels |
| --- | --- | --- | --- | --- |
| Human male | face | 0.22 | 0.98 | 417,957.00 |
|  | product | 0.35 | 0.86 | 136,018.20 |
|  | text | 0.88 | 0.32 | 738,487.80 |
| Human female | face | 0.27 | 0.91 | 417,957.00 |
|  | product | 0.62 | 0.51 | 136,018.20 |
|  | text | 0.87 | 0.32 | 738,487.80 |
| Virtual male | face | 0.22 | 1.16 | 417,957.00 |
|  | product | 0.38 | 0.66 | 136,018.20 |
|  | text | 0.88 | 0.32 | 738,487.80 |
| Virtual female | face | 0.20 | 0.95 | 417,957.00 |
|  | product | 0.40 | 0.65 | 136,018.20 |
|  | text | 0.76 | 0.43 | 738,487.80 |

Note. Mean luminance = mean CIE relative luminance Y within each AOI (range 0–1); RMS contrast = root-mean-square contrast within each AOI; Area pixels = number of pixels included in the AOI mask.

In designing the stimuli, we controlled the relative proportions of visual elements for each influencer. Accordingly, the AOIs were drawn so that the area of each AOI type (face, product, text) was kept constant across all advertisements.We report the average AOI pixels for each AOI type across the corresponding stimuli.

**TABLE S9** Means and standard deviations of key variables by influencer type.

| Type | *n* | Endorser TFD | Product TFD | Text TFD | Advertising attitude | Purchase intention |
| --- | --- | --- | --- | --- | --- | --- |
| Human male | 30 | 3.898 (1.630) | 7.618 (3.777) | 1.279 (1.118) | 3.820 (1.064) | 3.011 (0.837) |
| Human female | 30 | 4.612 (1.497) | 6.667 (2.566) | 3.322 (1.810) | 3.907 (0.975) | 3.322 (0.899) |
| Virtual male | 30 | 5.217 (2.813) | 5.383 (4.664) | 3.430 (1.847) | 3.360 (1.011) | 2.578 (0.788) |
| Virtual Female | 30 | 6.197 (2.820) | 4.558 (3.642) | 3.055 (2.251) | 3.547 (0.820) | 2.878 (0.837) |

**TABLE S10** Means, standard deviations, and correlations among study variables.

|  | *M* | *SD* | 1 | 2 | 3 | 4 | 5 | 6 |
| --- | --- | --- | --- | --- | --- | --- | --- | --- |
| 1.Influencer type | 0.500 | 0.502 | 1 |  |  |  |  |  |
| 2.Endorser TFD | 4.981 | 2.403 | 0.303** | 1 |  |  |  |  |
| 3.Product TFD | 6.056 | 3.873 | −0.281** | −0.017 | 1 |  |  |  |
| 4.Text TFD | 2.772 | 1.984 | 0.238** | −0.361** | −0.401** | 1 |  |  |
| 5.Advertising attitude | 3.658 | 0.984 | −0.209* | 0.053 | 0.108 | 0.066 | 1 |  |
| 6.Purchase intention | 2.948 | 0.873 | −0.252** | 0.080 | 0.121 | 0.097 | 0.792** | 1 |

Note. *N* = 120. **p* < 0.05, ***p* < 0.01

**
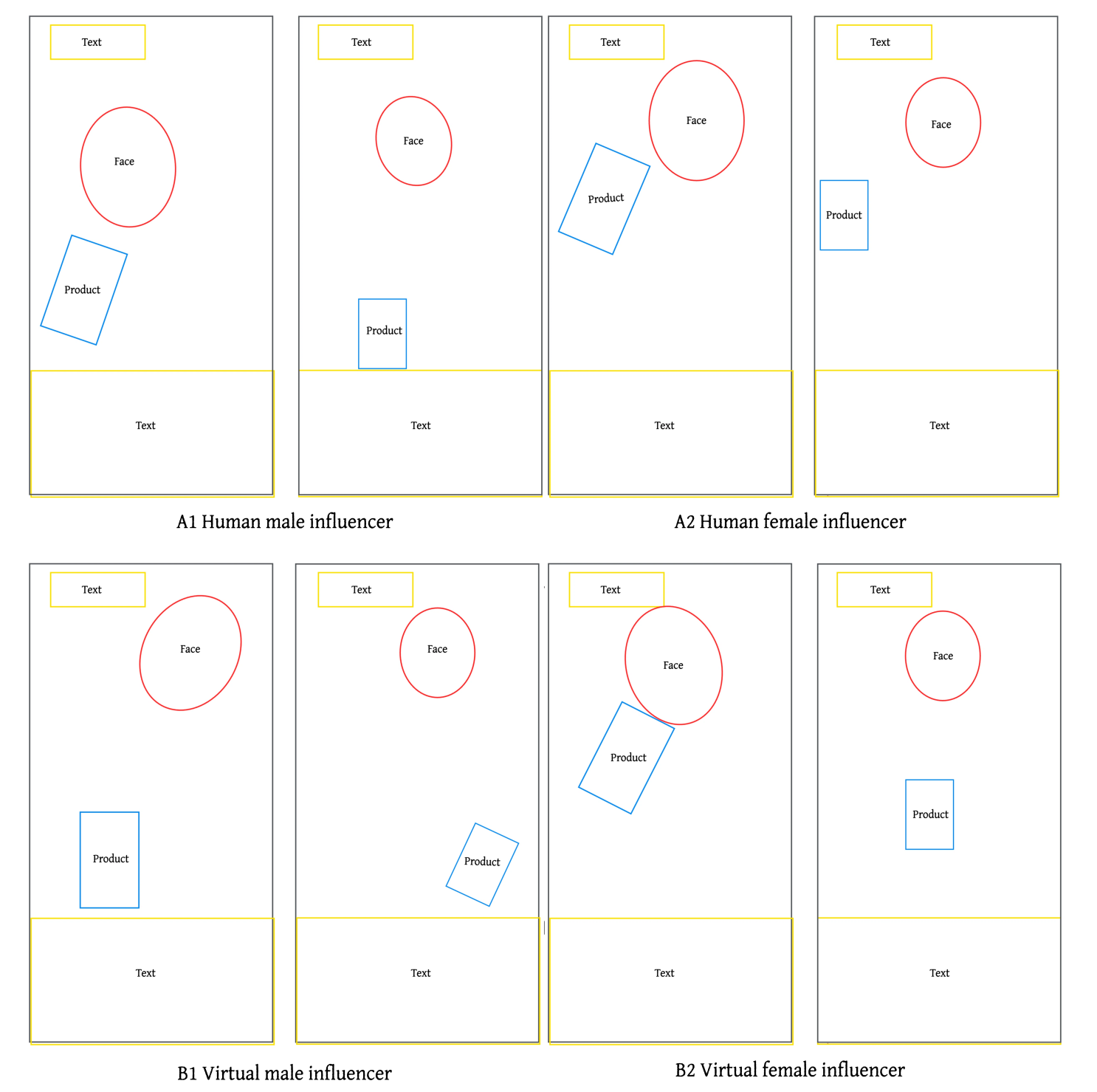
**

**FIGURE S1** Definition and location of AOIs in the stimuli.

Note. Face refers to the red elliptical area (model’s face), product to the blue rectangular area (product bottle), and text to the yellow rectangular area (profile information and advertising copy).


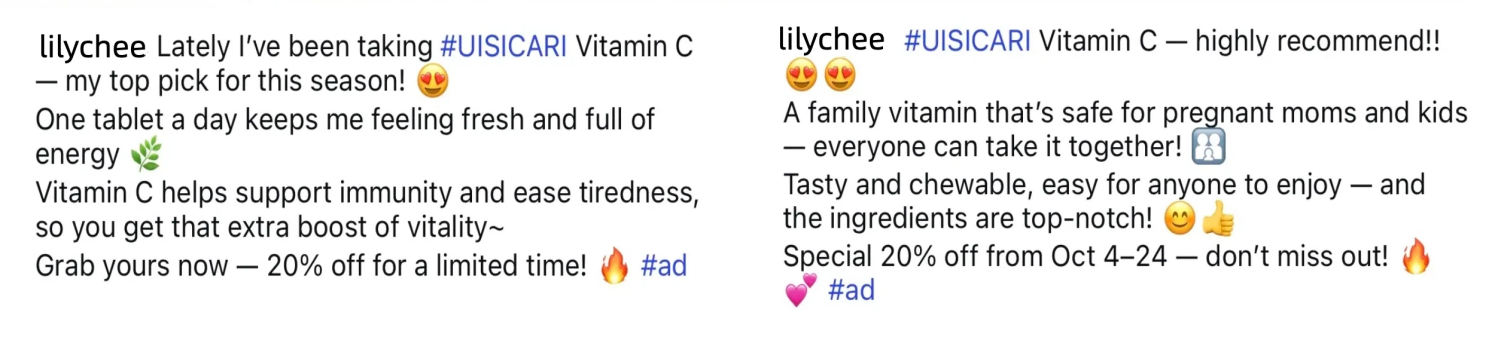


**FIFURE S2** Detailed advertising copy.
